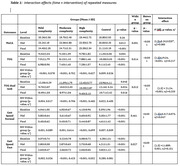# Effects of Different Intensity Exergame Balance Training on Balance and Cognition in Patients with MCI

**DOI:** 10.1002/alz.090844

**Published:** 2025-01-09

**Authors:** Aruba Saeed, Imran Amjad

**Affiliations:** ^1^ Riphah International University, Lahore, Punjab Pakistan; ^2^ Riphah International University, Islamabad, Punjab Pakistan

## Abstract

Integrating balance and cognitive training of varied intensities through exergame balance training may offer a distinct approach to enhancing balance and cognitive abilities in patients with mild cognitive impairment. The objective is to determine the relationship and effects of exergame balance training of different intensities on balance and cognition in patients with mild cognitive impairment (MCI). In this four‐arm parallel design Randomized Clinical Trial, ninety‐seven participants with mild cognitive impairments MoCA (18‐24), between the ages of 50 and 75 years, participated in novel exergame balance training. They were randomly assigned to one of four exergame balance training groups (mild, moderate, high, and control). All participants received twenty‐four sessions including forty minutes/sessions three times/week. The MoCA and BDNF were used to assess cognition, time up and go test (TUG), TUG dual task for functional mobility, Gait speed normal and fast for dynamic stability at baseline, after the 4th and 8th week. The correlation between cognition and balance was determined by spearman’s correlation. The mixed model analysis of covariance while fixing the baseline values as a covariate was used to determine interaction effects between interventions and time. Post hoc analysis was performed to investigate the findings between different complexity levels and the control group, and repeated measure ANOVA to determine within group differences.

There was a strong relationship between MoCA and TUG, TUG dual task with (r_s_ = 0.41), (r_s_ = 0.43) respectively and moderate relationship between MoCA and slow and Fast gait speed (r_s_ = 0.15), (r_s_ = 0.24) respectively of high intensity group. A significant interaction effect of group and time was observed for MoCA, TUG (1,3) = 19.34, <0.001, η2 = 0.217, TUG dual task (1,3) = 21.24, <0.001, η2 = 0.233, gait speed fast tests (1,3) = 11.889, 0.001, η2 = 0.151. Post‐hoc analysis at different intensities demonstrated a significant difference between mild, moderate and high complexity groups with the control group for MoCA, TUG, TUG dual task, Gait speed normal and fast (p<0.01).

The results indicate that exergame balance training of mild, moderate and high complexity influence the balance and cognition abilities differently with the improvement in the high intensity to a greater extent.